# Correlation of neck circumference with other anthropometric measurements among participants in a market survey at Ebonyi, South East, Nigeria

**DOI:** 10.11604/pamj.2025.50.71.45777

**Published:** 2025-03-12

**Authors:** Chidiebere Valentine Ugwueze, Michael Chinweuba Abonyi, Kenechukwu Emmanuel Onyekachi, Nnamdi Chukwunomso Anikpo, Chidiebele Malachy Ezeude, Collins Nwachi Ugwu, Nneka Marian Chika-Igwenyi, Thomas Obiora Nnaji, Basil Chukwuma Ezeokpo, Maris Unoma Ugwueze

**Affiliations:** 1 Department of Medicine, Alex-Ekwueme Federal University Ndufu- Alike Ikwo, Alex Ekwueme Federal University Teaching Hospital Abakaliki, Abakaliki, Ebonyi State,; 2 College of Medicine, Enugu State University, Enugu, Nigeria,; 3 College of Health Sciences, Nnamdi Azikiwe University Awka, Anambra State,; 4 Department of Medicine, Ebonyi State University, Abakaliki, Ebonyi State,; 5 Department of Nursing Sciences, David Umahi Federal University of Health Sciences, Uburu, Ebonyi State

**Keywords:** Neck circumference, obesity, anthropometric measurements, body mass index, waist circumference, hip circumference

## Abstract

**Introduction:**

neck circumference is one of the anthropometric measurements that are not routinely considered in the evaluation of patients with obesity and other non-communicable diseases (NCDs). This study was aimed at correlating neck circumference with other anthropometric measurements.

**Methods:**

it was a cross-sectional, descriptive study that involved the assessment of obesity among 197 participants (104 males and 93 males) using Body mass index (BMI), waist circumference, hip circumference, and neck circumference.

**Results:**

the male-to-female ratio was 1: 1: 1. The mean age of participants was 41.8±16.3 years. The prevalence of obesity among the participants was 17.8% which comprised (Class 1 obesity at 11.2%, class 2 obesity 5.1%, and morbid obesity at 1.5%). Using the waist-hip ratio (WHR), the prevalence of central obesity was 26.9% among the participants. The mean BMI, Waist circumference, WHR, and Neck circumference were 25.5±5.2, 88.1±14.1, 0.88±0.1, and 36.4±4.7 respectively. The mean neck circumference for males and females was 38.0cm and 34.5cm respectively. The mean neck circumference for males was significantly different from that of females (p< 0.001). The correlation of neck circumference with other measures of obesity was highly significant (p< 0.001) for BMI (r =0.29), waist circumference (r=0.80), hip circumference (r=0.85), and WHR (r= 0.34). The systolic and diastolic blood pressures correlated positively with neck circumference and all other measures of obesity with highly significant p-values.

**Conclusion:**

it is necessary to consider the use of neck circumference as a measure of obesity since it correlated significantly with other anthropometric measurements from the study. However, this is a one-centre study which limits the generalization of the findings.

## Introduction

Obesity is a chronic metabolic disease arising from excessive intake of calories in excess of consumption resulting in unhealthy and excessive fat deposition in different parts of the body. Obesity was initially considered a serious concern in developed nations of the world but unfortunately, it is becoming a worrisome condition in developing countries [1]. In 2022, about 43% of adults aged 18 years and above were overweight and 16% (890 million) persons were living with obesity according to the World Health Organization (WHO) fact sheet, 2024 [1]. Systematic studies in Nigeria reported the prevalence of obesity as ranging from 8.1 to 22.2 [2,3]. In sub-Saharan Africa, a comparative study which involved women of reproductive age showed that the prevalence of obesity was estimated as follows: South Africa (36%), Mauritania (27%), Eswatini (23%), Lesotho (20%), Gabon (19%) and Ghana (15%) [4]. A pooled study by Non-Communicable Disease Risk Factor Collaboration (NCD-RisC), Africa Working Group from 1980 to 2014 reported that the mean BMI increased from 21.0kg/m^2^ to 23.0kg/m^2^ in men and from 21.9kg/m^2^ to 24.9kg/m^2^ in women which describes an upward trend [5]. There was an increased BMI in all the regions involved in the study. Though obesity was initially regarded as a manifestation of healthy living [6,7], there are many complications arising from obesity including hypertension, diabetes mellitus, osteoarthritis, poor wound healing, respiratory diseases, and cancers [8,9]. Due to the multiple effects of obesity, the latter is considered to have a public health importance. In the evaluation of obesity, various anthropometric measurements are in use for sometimes and they include: body mass index, waist circumference (WC), hip circumference (HC), and waist-to-hip ratio [10,11]. Neck circumference (NC) is among the newer measures being considered [12].

Neck circumference as a measure of obesity is easier and may not necessarily involve exposing a larger part of the body. Neck circumference as a measure of obesity is easily adapted to the patient as it is less cumbersome. Neck circumference is an indicator of subcutaneous fat in the upper regions of the body and also a predictor of cardiovascular risk as reported by Kim *et al*., and Ataie -Jafari *et al*. [13,14]. Many authors have reported a strong correlation between neck circumference and waist circumference in patients with prediabetes and diabetes [12,15,16]. A review of anthropometric indices among 133 healthy persons by Omozehio *et al*. [17] showed a significant positive correlation between neck circumference and WC, HC, and BMI (Pearson´s correlation, r=0.62, 0.65, 0.66 respectively). Similar findings were obtained by other authors [18,19]. The correlation of neck circumference (NC) with WC, HC, and BMI was stronger in males than in females [17,20]. The mean neck circumference in different studies were: 40.45 ± 2.68 cm for men and 32.71 ± 2.37 cm for females [20], 38.9 ± 3.2 cm for men and 35.4 ± 3.1 cm [21], 38.9 cm for men and 36 cm for women [22]. Sruthi *et al*. [23] opined that among Indians, a neck circumference of more than 35.5cm in men and more than 32 cm in women should be considered obesity. High neck circumference is a predictor of hypertension and obstructive sleep apnea as revealed by Ren *et al*. [24] and Cao *et al*. [25] respectively. Furthermore, in assessment of insulin resistance, neck circumference was shown to be a better anthropometric parameter than waist circumference [26-28]. It has also been demonstrated that NC was a better predictor of glucose homeostasis and HDL-Chol concentrations when compared to BMI and WC [28]. This study was set out to determine the prevalence of obesity and the correlation between neck circumference and BMI, waist circumference, hip circumference, and waist-to-hip ratio among participants.

## Methods

**Study design and setting:** it is a cross-sectional and descriptive study. The study was carried out at Margret Umahi International Market, Abakaliki, Ebonyi State. It is the most prominent market in Ebonyi State and attracts traders from adjoining States such as Cross River State.

**Study population:** the study participants consisted of 197 individuals who consented to the study. The participants were mainly traders and other individuals of different professions who attended the market on the day of the study.

**Inclusion criteria:** i) individuals aged above 18 years and; ii) individuals who gave consent to participate in the study.

**Exclusion criteria:** i) individuals who did not consent to the study; ii) those who are pregnant.

**Sampling technique:** convenience sampling technique was used as participants were recruited as they presented to the study venue.

**Procedure and data collection:** the weight of participants was measured with the weighing scale of a stadiometer (Seca; Steindham, Hamburg- Germany, 2013). The stadiometer was used to obtain the height. Body mass index was calculated as weight in kilograms divided by height in meters squared (kg/m^2^). The neck, waist circumference, and hip circumference of participants were measured with a stretchable measuring tape.

**Neck circumference:** a stretchable measuring tape was placed on a horizontal plane at the lower edge of the cricoid cartilage while the participant was to look forward directly in front [20]. A measuring tape is wound around the neck between the mid-anterior neck (the most prominent portion of thyroid cartilage) and mid-cervical spine to obtain the neck circumference [20]. The waist circumference was obtained with a stretchable measuring tape wound around the midway between the last palpable rib and iliac crest [29]. The hip circumference was obtained as the widest circumference around the gluteus maximus.

**Statistical analysis:** the data obtained from the study was analyzed using the Statistical Package for Social Sciences SPSS (IBM-SPSS) for Windows version 23 (IBM, Corp., Armonk N.Y., USA). The characteristics of participants were summarized using descriptive statistics. The prevalence was calculated as frequencies and percentages as shown in Table 1. Pearson´s correlation was used to assess the correlation between neck circumference and other anthropometric measurements as shown in Table 2. Similarly, the correlation of blood pressure with different anthropometric measurements involved the use of Pearson´s correlation. A significant correlation was defined by p-values ≤ 0.05. Student- t-test was used to compare the mean neck circumference of male and female participants as in Table 3.

**Table 1:** prevalence of obesity among participants

Variable	Frequency n=197	Percent
Body weight (WHO* classification)		
Underweight	6	3.0
Normal weight	89	45.2
Overweight	67	34.0
Class 1 obesity	22	11.2
Class 2 obesity	10	5.1
Morbid obesity	3	1.5
Waist circumference (IDF**)		
Normal (male: <94cm or female: <80cm)	98	49.7
Centrally obese (male: ≥94cm or female: ≥80cm)	99	50.3
**Waist-hip ratio (WHO classification**)		
Below moderate	105	53.3
Moderate (male: 0.96 - 1.0 or female: 0.81 - 0.86)	39	19.8
High (male: >1.0 or female: ≥0.86)	53	26.9

WHO; World Health Organization; IDF: International Diabetes Federation

**Table 2:** correlation of neck circumference with standard obesity measurements

Variable	N	Bivariate correlation		
		Pearson’s r	P-value	95% Confidence interval
Body mass index	197	0.291	<0.001	0.158 - 0.493
Hip circumference	197	0.846	<0.001	0.115 - 0.458
Waist circumference	197	0.804	<0.001	0.127 - 0.531
Waist-hip ratio	197	0.337	<0.001	0.051 - 0.378

**Table 3:** comparison of mean neck circumference of males with that of females

Variable	N=197	Mean	Standard deviation	Student-t	P-value
**Sex**					
**Female**	93	34.5054	5.67723	5.5999	<0.001
**Male**	104	38.0192	2.79730		
**Total**	197	36.3604	4.72586		

**Ethical consideration:** an ethical clearance for the study was obtained from the Ethics and Research Committee of the Alex-Ekwueme Federal University Teaching Hospital, Abakaliki with an Ethical Clearance number: AE-FUTHA/REC/VOL3/2022/087. Informed consent was obtained from the participants and were allowed to withdraw at any time in the study without any negative impact on their treatment. This study adhered to the STROBE guidelines for observational research, ensuring transparency and robustness in study design, data collection, and reporting.

## Results

**Demographic characteristics:** the study participants were 197: 104 males and 93 females. The participants were traders (51.8%), civil servants (18.3%), farmers (8.6%), drivers (5.6%), students (4.6%), health workers (2.5%) and others (8.6%) respectively. The average age of the subjects was 41.8 ± 16.3 years.

**Prevalence of obesity:** the prevalence of obesity among the participants was 17.8% which comprised of (class 1 obesity 11.2%, class 2 obesity 5.1%, and morbid obesity 1.5%) as shown in Table 1. Using the waist-hip ratio (WHR), the prevalence of central obesity was 26.9% among the participants (Table 1). Correlation of neck circumference and other parameters. The correlation of neck circumference with other measures of obesity was highly significant: for BMI (p< 0.001, r =0.291), waist circumference (p< 0.001, r= 0.804), hip circumference (p < 0.001, r= 0.846) and WHR (p< 0.001, r=0.337) as shown in Table 2. The mean of different anthropometric measurements was: BMI (25.5 ±5.2 kg/m^2^), waist circumference (88.1±14.1 cm), hip circumference (100.4±11.5), waist - hip ratio (0.88 ± 0.1) and neck circumference (36.4 ± 4.7 cm). The mean neck circumference for males and females was 38.0cm and 34.5cm respectively. The mean neck circumference for males was significantly different from that of females (p< 0.001). as shown in Table 3. The systolic and diastolic blood pressures correlated positively with neck circumference and all other measures of obesity with highly significant p-values (Table 4).

**Table 4:** correlation of blood pressure with standard obesity measurements

Standard obesity measurements	Systolic BP n=197		Diastolic BP n=197	
	Pearson’s r	P-value	Pearson’s r	p-value
Neck circumference	0.196	0.006	0.145	0.041
Hip circumference	0.231	0.001	0.185	0.009
Waist circumference	0.299	<0.001	0.228	0.001
Waist-hip ratio	0.230	0.001	0.169	0.018
Body mass index	0.208	0.003	0.166	0.020

BP: blood pressure

## Discussion

The study aimed at determining the prevalence of obesity as well as correlating neck circumference with other anthropometric parameters. The results revealed the prevalence of overweight and obesity as 34% and 17% respectively (Table 1). The prevalence of obesity obtained using waist circumference was higher than that of BMI. Moreover, the neck circumference correlated significantly and positively with other anthropometric parameters. The implication of the prevalence of overweight and obesity from this study is that it is a common metabolic problem. The study population consisted of mainly traders who may not adhere to routine healthy eating and physical exercise regimens, thus giving rise to a high prevalence of obesity. This is similar to the finding of a systematic review by Chukwunonye *et al*.[2] who reported the prevalence of overweight among Nigerian men and women as 26.3% and 28.3% respectively while the prevalence of obesity was 10.9% and 23.0% respectively. Similarly, a higher prevalence of obesity obtained using waist circumference compared to BMI has also been substantiated by Shrestha *et al*. [30]. Central obesity is usually considered to have poorer health outcomes than generalized obesity [31]. This is because of the degree of inflammation initiated by central adiposity. Thus, the use of waist circumference to determine central obesity is usually adopted by most researchers rather than BMI. The limitation of BMI is the difficulty in distinguishing weight contributed by muscles, fats, and bone density [32]. The neck circumference from this study was shown to have a significantly positive correlation with all the other standard obesity measurements (Table 2). The reason for the positive correlation is due to the corresponding accumulation of subcutaneous fat in the neck as fats also accumulate in other areas of the body in obese patients. This is similar to the findings of Omozehio *et al*. and Qureshi *et al*. [17,19]. Omozehio *et al*. [17] demonstrated a significantly positive correlation between neck circumference and other anthropometric parameters such as waist circumference, hip circumference, and BMI.

The mean NC of males and females in this study was 38 cm and respectively as shown in Table 3. The finding is similar to the findings of Olusola *et al*. [22] in South Western, Nigeria. However, there was a slight difference between the findings in this study and that of Ukoha *et al*. [20] even though higher values were still noted in males. The predominantly higher NC in males compared to females has been substantiated in many studies [33,34]. The gender difference is related to the soft tissue mass and fat deposits in the neck which has been shown to be higher in males than in females using magnetic resonance imaging [35]. Men with NC > 37 cm and women with NC > 34 cm are more prone to cardio-metabolic syndrome and require more evaluation [36]. The correlation of blood pressure with neck circumference and other obesity measurements was significant as shown in Table 4. This was also similar to the findings of Ren *et al*. [24] who demonstrated that higher neck circumference predicted hypertension in about 42 % of study subjects. Alfadhli *et al*. [37] also demonstrated the positive correlation of neck circumference with systolic blood pressure in both males and females and between NC and diastolic blood pressure in men but not in women. Similarly, Ramoshaba *et al*. [38] have equally demonstrated among 127 students aged 18 years and above that NC significantly correlates with both systolic (r=0.5; p< 0.001) and diastolic blood pressures (r=0.3; p < 0.001) using Pearson´s correlation analysis. Makaju *et al*. [39] in a study among 1000 Nepalese patients attending Manipal Teaching Hospital showed a similar correlation of NC with both systolic and diastolic blood pressure but the correlation was more positive with diastolic blood pressure among female participants taking antihypertensive medication.

The explanation for the correlation of neck circumference and blood pressure is that central obesity as reflected by increased neck circumference gives rise to the release of some inflammatory cytokines such as tumor necrosis factor-α and IL-6. The cytokines can cause vascular endothelial dysfunction leading to increased vascular resistance and elevated blood pressure [40]. Moreover, obesity as manifested by increased neck circumference brings about an increase in sympathetic nervous system activity which elevates heart rate, total peripheral resistance, and blood pressure [41]. The limitation of this study is that it is a one-centre study which may limit the validation of the findings and will require multiple-centre studies for better validation. The conducting of this study in a market area makes the study population a good representation of the society. The correlation of neck circumference with more than one anthropometric measurement adds strength to the study.

## Conclusion

The importance of neck circumference as a measurement for obesity assessment cannot be over-emphasized. The significant correlation of NC with other anthropometric measurements makes it very reliable and efficient in the management of patients with obesity. Furthermore, the correlation of neck circumference with blood pressure makes former an invaluable tool in the management of hypertension and obesity.

### What is known about this topic

- Obesity is one of the cardio-metabolic diseases;
- Anthropometric measurements used in assessing obesity in most studies are body mass index (BMI) and waist circumference and waist-hip ratio.

### What this study adds

- The study demonstrated the relevance of neck circumference in obesity assessment;
- The significant correlation of neck circumference with other anthropometric measurements emphasizes the importance of neck circumference in obesity evaluation;
- Moreover, the study also demonstrated significant correlation of neck circumference with systolic and diastolic blood pressures.

## References

[1] World Health Organization (2024). Obesity and overweight. WHO.

[2] Chukwunonye II, Ohagwu KA, Ogah OS, John C, Oviasu E, Anyabolu EN (2022). Prevalence of overweight and obesity in Nigeria: systematic review and meta-analysis of population-based studies. PLOS Glob Public Health.

[3] Adeloye D, Ige-Elegbede JO, Ezejimofor M, Owolabi EO, Ezeigwe N, Omoyele C (2021). Estimating the prevalence of overweight and obesity in Nigeria in 2020: a systematic review and meta-analysis. Ann Med.

[4] Owobi OO, Okonji OC, Nzoputam CI, Ekholuenetale (2022). Country-level variations in overweight and obesity among reproductive-aged women in sub-Saharan countries. Women.

[5] Kegne AP, Bentham J, Zhou B, Peer N, Matsha TE, Bixby H (2017). Trends in obesity and diabetes across Africa from 1980 to 2014: an analysis of pooled population-based studies. Int J Epidemiol.

[6] Alexander E, Sewyn A, Calitz C, Yach D, Wang YC (2016). Obesity, causes, and prevalence. Encyclopedia of Food and Health.

[7] Osayomi T (2020). Being fat is not a disease but a sign of good living. The political economy of overweight and obesity in Nigeria. Ghana J Geography.

[8] Piche ME, Tchernorf A, Despres JP (2020). Obesity phenotypes, diabetes, and cardiovascular diseases. Circ Res.

[9] Ashraf MJ, Baweja P (2013). Obesity: the ‘huge´ problem in cardiovascular diseases. Mo Med.

[10] World Health Organization (2010). A healthy lifestyle-WHO recommendations. WHO.

[11] World Health Organization (2008). Waist circumference and waist-hip ratio: report of a WHO expert consultation. WHO.

[12] Anothaisintawee T, Sansanayudh N, Thamakaison S, Lertrattananon D, Thakkinstian A (2019). Neck circumference as an anthropometric indicator of central obesity in patients with prediabetes: cross-sectional study. Biomed Res Int. 2019 Jun 9.

[13] Kim KY, Moon HR, Yun JM (2021). Neck circumference as a predictor of metabolic syndrome in Koreans: A cross sectional study. Nutrients.

[14] Ataie-Jafari A, Namazi N, Djalalinia S, Chaghamirzayi P, Abder ME, Zadehe SS (2018). Neck circumference and its association with metabolic risk factors: A systematic review and meta-analysis. Diabetol Metab Syndr.

[15] Aswthappa J, Garg S, Kutty K, Shankar V (2013). Neck circumference as an anthropometric measure of obesity in diabetics. N Am J Med Sci.

[16] Wang X, Zhang N, Yu C, Ji Z (2015). Evaluation of neck circumference as a predictor of central obesity and insulin resistance in Chinese adults. Int J Clin Exp Med.

[17] Omozehio IS, Ahmad SA, Adeyemi OM, Adetola FO, Efedaye OA (2014). The relationship between neck circumference and other indices of adiposity in a healthy Nigerian population. Nig Quarterly J Hospital Medicine.

[18] Tantawy SA, Kamel DM, Alsayed N, Rajab E, Abdelbasset WK (2020). Correlation between body mass index, neck circumference, and waist-hip ratio as indicators of obesity among a cohort of adolescent in Bahrain: a preliminary cross-sectional study. Medicine (Baltimore).

[19] Qureshi NK, Hossain T, Hassan MI, Akter N, Rahman NN, Sultana MM (2017). Neck circumference as a marker of overweight and obesity and cutoff values for Bangladeshi Adults. Indian J Endocrinol Metab.

[20] Ukoha UU, Ekezie J, Ukoha C, Obazie IF (2018). Study of Neck circumference as a measure of obesity in South Eastern Nigerian population. Anthropol Open.

[21] Mohseni-Takallo S, Mozaffari-Khosravi H, Mohseni H, Mirzaei M, Hosseinzadeh M (2023). Evaluating neck circumference as an independent predictor of metabolic syndrome and its components among adults: A population-based study. Cureus.

[22] Olusola DA, Mumani AA, Olajumoke OB (2023). Neck circumference and cardiometabolic syndrome in adult patients at a tertiary hospital in Lagos, Nigeria: A cross sectional study. J Clin Sci.

[23] Sruthi KG, John SM, David SM (2023). Assessment of obesity in Indian setting: A clinical review. Clin Epidemiol Glob Health.

[24] Ren TJ, Zhang K, Li WJ, Ren ST, Huang YZ, Yang N (2023). Body mass index, neck circumference and hypertension: a prospective study. Front Cardiovasc Med.

[25] Cao MT, Guilleminault C, Kushida CA (2011). Clinical features and evaluation of Obstructive Sleep apnea and upper airway resistance syndrome. Principles and Practice of Sleep Medicine.

[26] Vatier C, Poitou C, Clement K (2014). Evaluation of visceral fat in massive obesity. Medicine.

[27] Yang L, Samarasinghe YP, Kane P, Amiel SA, Aylwin SJB (2010). Visceral adiposity is closely correlated with neck circumference and represents a significant indicator of insulin resistance in WHO grade III obesity. Clin Endocrinol (Oxf).

[28] Scoovronec A, Provencher A, Iceta S, Pelletier M, Leblanch V, Nadeau M (2022). Neck circumference is a better correlate of insulin resistance markers than other standard anthropometric indices in patients presenting with severe Obesity. Obes Res Clin Pract.

[29] Gibson RS (1990). Anthropometric assessment of body composition. Principles of Nutritional.

[30] Shrestha R, Upadhyay SK, Khatri B, Bhattarai JR, Kayastha M, Upadhyay MP (2021). BMI, waist to height ratio and waist circumference as a screening tool for hypertension in hospital outpatients: a cross-sectional, non-inferiority study. BMJ Open.

[31] Lempesis IG, Georgakopoulou VE (2023). Physiopathological mechanisms related to inflammation in obesity and type 2 diabetes. World J Exp Med.

[32] Buss J (2014). Limitations of body mass index to assess body fat. Workplace Health Saf.

[33] Zigova M, Miriama S Vaskova H, Jana G (2023). Confirmation of significant correlations between neck circumference and anthropometric indicators of cardio-metabolic health in the group of university students in Eastern Slovakia. Ceska Antropologie.

[34] Lucas RE, Fonseca ALF, Dantas RO (2016). Neck circumference can differentiate obese from non-obese individuals. Medical Express.

[35] Whittle AT, Marshall I, Mortimore IL, Wraith PK, Sellar RJ, Douglas NJ (1999). Neck soft tissue and fat distribution: comparison between normal men and women by magnetic resonance imaging. Thorax.

[36] Kumar NV, Ismail MH, Mahesha P, Girish M, Tripathy M (2014). Neck circumference and cardio-metabolic syndrome. J Clin Diagn Res.

[37] Alfadhli EM, Sandokji AA, Zahid BN, Makkawi MA, Alshenaifi RF, Thani TS (2017). Neck circumference as a marker of obesity and a predictor of cardiometabolic risk among Saudi subjects. Saudi Med J.

[38] Ramoshaba NE, Fihla MQ, Mthethwa WS, Tshangela L, Mampofu ZM (2022). Neck circumference and blood pressure measurements among Walter Sisulu University students. Int J Environ Res Public Health.

[39] Makaju S, Chaudhary S, Alam M (2018). Correlation of the neck circumference with blood pressure. J Coll Med Sci.

[40] dos Passos RR, Santos CV, Priviero F, Briones AM, Tostes RC, Webb RC (2024). Immunomodulatory activity of cytokines in hypertension: a vascular perspective. Hypertension.

[41] Valensi P (2021). Autonomic nervous system changes in patients with hypertension and overweight: role and therapeutic implications. Cardiovasc Diabetol.

